# Nucleocapsid-like cryo-EM structure of influenza B virus

**DOI:** 10.1371/journal.ppat.1014449

**Published:** 2026-08-10

**Authors:** Marie Thirion, Alice J. Stelfox, Florian Chenavier, Héléna Chomat, Catherine Isel, Lily-Lorette Freslon, Eleftherios Zarkadas, Rob W. H. Ruigrok, Nadia Naffakh, Thibaut Crépin, Allison Ballandras-Colas

**Affiliations:** 1 Université Grenoble Alpes, CNRS, CEA, IBS, Grenoble, France; 2 RNA Biology and Influenza Viruses, Institut Pasteur, Université Paris Cité, CNRS UMR3569, Paris, France; 3 Université Grenoble Alpes, CNRS, CEA, EMBL, ISBG, Grenoble, France; University of Zurich: Universitat Zurich, SWITZERLAND

## Abstract

Influenza viruses belong to the *Orthomyxoviridae* family, they are categorized into four types: A, B, C, and D. Influenza B viruses co-circulate annually with influenza A strains during seasonal flu epidemics in humans, causing severe disease. The segmented RNA encapsidated by multiple copies of the nucleoprotein (NP) and attached to the heterotrimeric polymerase forms the central replicative unit called the viral ribonucleoprotein (vRNP). All influenza NP proteins share the same core domain folding, only NP of influenza B virus (B/NP) has an extended unfolded N-terminal tail of 70 amino-acids. This disordered N-terminal tail is required for nuclear localization of the protein, and may be involved in viral RNA transcription and replication regulation. In this study, we report that in absence of the extended N-terminal tail, the truncated NP maintains RNA binding ability and NP oligomeric state *in vitro*. We then reconstituted RNP-like particles by incubating truncated B/NP with synthetic RNA and we solved the cryo-EM structure at 4.1 Å resolution. Their morphology appears identical to native vRNPs extracted from viruses when observed by negative-stain electron microscopy. Overall, our results suggest that vRNPs from influenza virus type A, B and D share the same right-handed antiparallel helical conformation and that the B/NP N-terminal tail does not participate to the helical architecture stabilization once the vRNPs are assembled.

## Author summary

Influenza virus A and B circulate every winter and cause severe human disease during seasonal flu outbreaks. The RNA genome of influenza virus is segmented and encapsidated by the nucleoprotein (NP) and, with the heterotrimeric polymerase, form the viral ribonucleoprotein (vRNP). vRNP are central to viral replication, understanding how they function is essential to decipher influenza virus biology. NP protects the viral RNA and oligomerize into large helicoidal assembly. Influenza A and B NPs are highly conserved, despite belonging to separate type of the *Orthomyxoviridae* family. They both fold into a compact core domain, however, B NP has an additional N-terminal extension, which function is not clearly understood. In this study, we report that the extended N-terminal tail does not impair RNA binding ability and NP oligomeric state *in vitro.* We then reconstituted the vRNP lacking the polymerase *in vitro*, and report the cryo-EM structure at 4.1 Å resolution that displays similar helical arrangement with vRNPs from influenza A and D.

## Introduction

Influenza, commonly referred to as the flu, is a highly contagious respiratory illness caused by influenza viruses. They represent a major public health challenge as influenza viruses cause recurrent seasonal epidemics and occasional pandemics. Indeed, the highly pathogenic avian H5N1 virus, which has already caused a large on-going outbreak in dairy cows in the United States [1–4], may further evolve to enable more efficient human-to-human transmission [5], potentially resulting in a severe pandemic.

Influenza viruses belong to the *Orthomyxoviridae* family with a negative-sense, single stranded genome, and are classified into four types: A, B, C, and D, based on NP antigenic differences. Among them, influenza A and B are the primary causes of seasonal epidemics in humans.

Influenza A is the most genetically diverse of influenza viruses. Wild aquatic birds constitute the primary reservoir of genetic diversity for influenza A viruses, from which they can infect a wide range of other animal species, both wild and domestic, avian and mammalian, including humans. In contrast, influenza B primarily affects humans and does not have animal reservoirs, which limits its potential to cause pandemics [6]. However, it contributes significantly to seasonal epidemics, often leading to severe illness, especially in children and immunocompromised individuals [7]. Despite these differences, influenza viruses share similar morphology and life cycle.

The genome of influenza A and B viruses is divided into eight segments, which are packaged as separate viral ribonucleoprotein (vRNP) complexes in the viral particle. Each vRNP consists of a single heterotrimeric RNA-dependent RNA polymerase (FluPol) attached to the conserved and partially complementary 3′ and 5′ RNA ends, the rest of the vRNA is covered with multiple copies of the viral nucleoprotein (NP). NP encapsidates the viral RNA segments (vRNA) in a non-uniform and non-random manner, albeit without sequence specificity [8–13]. The vRNP forms an elongated and flexible structure, folded into a loose helical filament with two antiparallel strands. vRNPs constitute the central functional unit for transcription and replication of the viral genome within the host cell [14,15]. Important studies have highlighted crucial details of the molecular mechanism of transcription and RNA replication using high-resolution structures of FluPol (reviewed in [16]). In contrast, cryo-EM structures of vRNPs have been limited to nanometric resolution due to their inherent flexibility [17–19], hindering the detailed visualization of the viral RNA encapsidated by the NPs or of the FluPol attachment to the nucleocapsid (NP-RNA part of the vRNP). Recently, the cryo-EM analysis of a A/mini-RNP generated using a mini-replicon system, based on strategy established in Juan Ortin’s lab [20], revealed the interactions between the A/FluPol and A/NP-RNA complex in the context of a short ring-like RNP [21]. Previously, we established a protocol to reconstitute recombinant nucleocapsid-like particles *in vitro* by incubating purified full-length A/NP (A/WSN/1933) with a short synthetic ^5′^P-(UC)_6_-fluorescein^3’^ RNA (^5′^P-(UC)_6_-FAM^3’^, called 12-mer for clarity) [22,23]. The subsequent cryo-EM structure, obtained at sub-nanometer resolution, allowed for the first time the visualization of both A/NP-A/NP and A/NP-RNA interactions organized in a right-handed parallel double stranded helical particle. This nucleocapsid-like structure enabled the placement of the RNA backbone at the NP-NP interface, and revealed the several structural rearrangements A/NP must accommodate to oligomerize in a helical structure and to bind the RNA. Subsequent to this study, we further optimized the reconstituted nucleocapsid-like strategy by partially truncating the disordered N-terminal extremity of A/NP and modulating the RNA length [24]. We thus obtained an improved right-handed double-stranded antiparallel helical cryo-EM structure at 3.0 Å resolution. This structure reveals (i) the presence of major and minor grooves along the antiparallel helical particle, (ii) that A/NP-A/NP interactions are solely responsible for the helical inter-strand contacts, (iii) that using a ^5′^P-(UC)_9_-FAM^3’^ (18-mer) the RNA follows an almost continuous path across all A/NPs, and (iv) that the RNA EM density is the least well resolved highlighting the high degree of RNA mobility. Very recently, the highest resolution structure, so far, of a helical vRNP was published at 8.6 and 5.1 Å resolution, corresponding to the global and focused map, respectively [25]. It was obtained using the shortest segment – the Non-Structural (NS) segment – of the influenza D virus, generated by using a mini-replicon system and revealed a right-handed double stranded antiparallel helical structure with the characteristic major and minor groove. Although the nucleocapsid-like (or A/RNP-like) structure is slightly more compacted compared to that of the D/vRNP, notably due to the presence of inter helical-strand contacts and tighter NP-NP interfaces along one helical-strand, both structures are highly similar in the overall organization, especially regarding the RNA pathway and NP loops remodeling to accommodate the RNA. Altogether, the D/vRNP structure validates the A/RNP-like structure is a relevant model of the vRNP to describe the molecular basis of RNA encapsidation.

In the absence of a reported B/vRNP structure, we investigated the relevance of these findings to influenza B by reconstituting the B/nucleocapsid-like particle and analyzing the resulting EM structure.

## Results

### Impact of the long N-terminal tail of B/NP on RNA binding and oligomeric state

**Fig 1:**
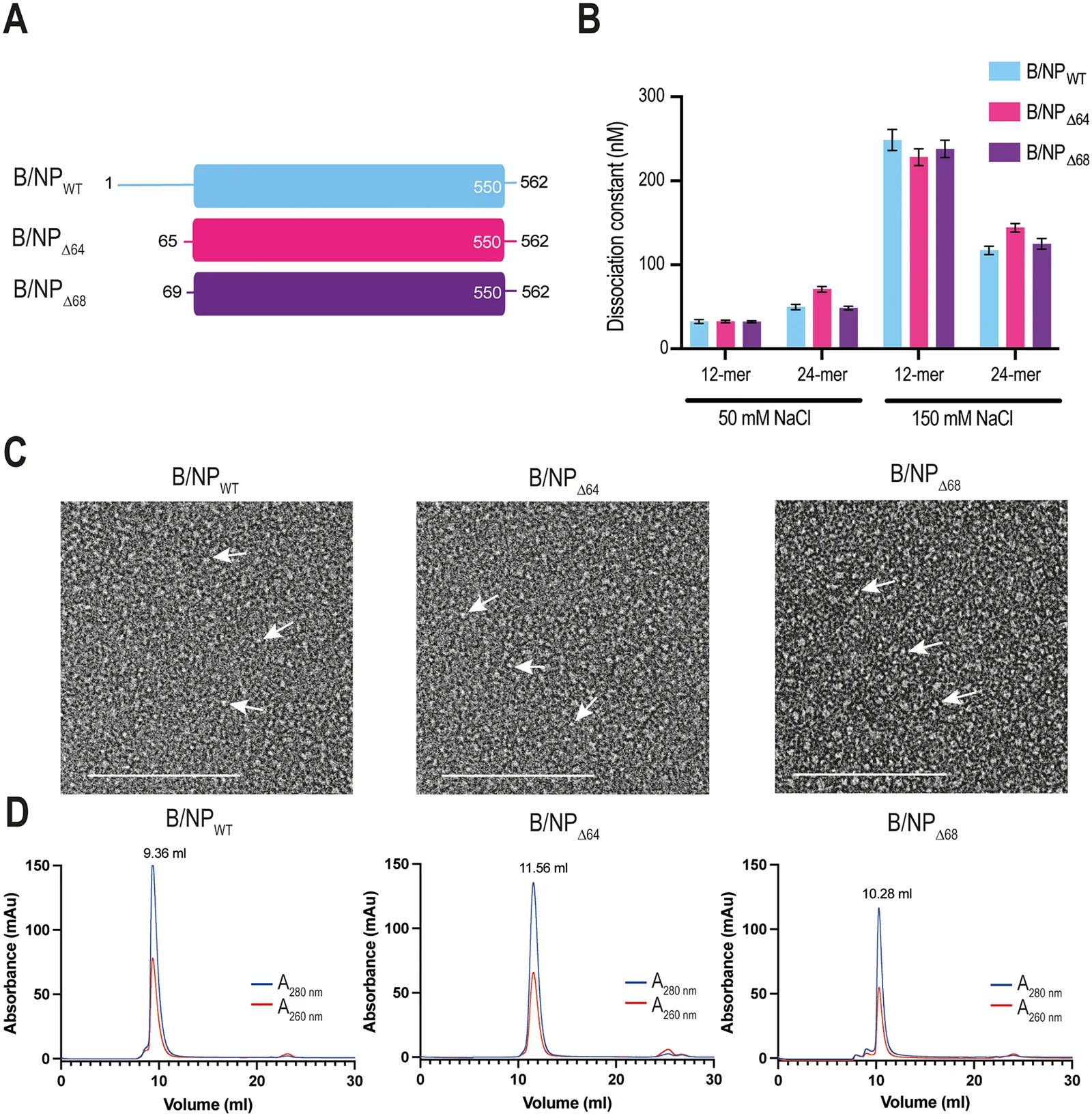
Recombinant B/NP_WT_, B/NP_∆64_ and B/NP_∆68_ RNA affinity and oligomeric state. **A.** Schematic representation of B/NP_WT_, B/NP_∆64_ and B/NP_∆68_ in light blue, pink and purple, respectively. The rectangle represents the folded core of the protein. **B.** Fluorescence anisotropy measurements to quantify B/NP_WT_, B/NP_∆64_ and B/NP_∆68_ affinity for the 12-mer or 24-mer synthetic RNAs, in 50 or 150 mM NaCl. Color coding consistent with **A**. **C.** Visualization by electron microscopy with negative staining of B/NP_WT_, B/NP_∆64_ and B/NP_∆68_ purified in 50 mM NaCl buffer. Arrows points at monomeric protomers. Scale bars represent 100 nm. **D.** Size exclusion chromatography profiles of B/NP_WT_, B/NP_∆64_ and B/NP_∆68_ eluted in the same buffer with 50 mM NaCl.

B/NP shares the 3D folding common to all influenza NPs, comprising a head domain, a body domain, a central basic groove and an oligomerization loop. However, unlike A/NP, B/NP has a 70 amino acid N-terminal disordered tail, whereas A/NP has a shorter 20-residues N-terminal tail [26,27]. Given that truncating the first 14 residues of A/NP N-terminal tail enabled the formation, *in vitro*, of a double-stranded antiparallel helical nucleocapsid-like A/RNP-like particle, we designed two constructs derived from B/Memphis/13/2003 NP in which the first 64 or 68 amino acids were removed, respectively, generating NPs with residues 65–562 (B/NP_∆64_) and 69–562 (B/NP_∆68_) of the wild-type NP (B/NP_WT_) protein (Figs 1A and S1). We expressed and purified the wild type protein as well as the truncated constructs. First, we assessed their ability to bind RNA with fluorescence anisotropy measurements using short synthetic RNA probes, ^5′^P-(UC)_6_-FAM^3’^ (12-mer) and ^5′^P-(UC)_12_-FAM^3’^ (24-mer) in different NaCl conditions (Fig 1B). Affinity measurements in a buffer containing 50 mM NaCl indicate Kd values of 30–70 nM for both 12- and 24-mer RNA probes with all three constructions, while at 150 mM NaCl, the affinity constants rise to 240 and 120 nM for the 12 and 24-mer RNAs, respectively. These results reveal that all three constructs share comparable affinity to the RNA probes independent of RNA length or buffer conditions. Next, we established the oligomeric state of B/NP_WT_, B/NP_∆64_ and B/NP_∆68_ in solution using size exclusion chromatography at 50 mM NaCl, coupled with visualization by negative staining electron microscopy (NS-EM). Although the SEC elution volumes shift, images recorded by NS-EM confirm that all three proteins are largely monomeric under these conditions (Fig 1C and 1D). Altogether, these findings demonstrate that the unfolded N-terminal tail has no effect on RNA binding nor on the protein oligomeric state.

### B/NP ability to form RNP-like particles *in vitro*

**Fig 2:**
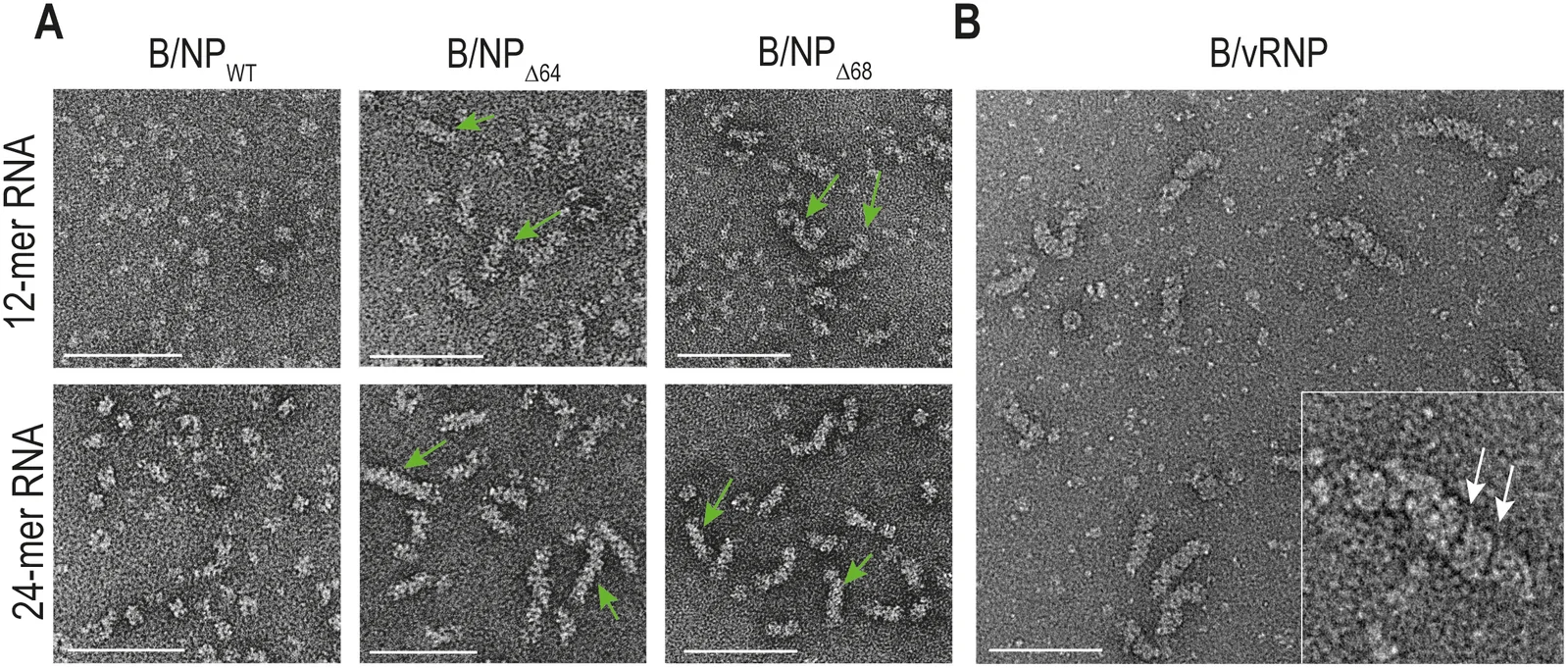
Visualization of RNP-like particles and vRNP. **A.** Observation by electron microscopy with negative staining of B/NP_WT_, B/NP_∆64_ and B/NP_∆68_ incubated with the 12-mer or 24-mer synthetic RNA. The formation of RNP-like filaments is indicated with green arrows. **B.** B/vRNP extracted from viruses observed by electron microscopy with negative staining. Bottom right, close up view of one B/vRNP, with white arrows pointing to the major groove. Scale bars represent 100 nm.

In our previous work, we optimized a method that allows the formation of double stranded antiparallel RNP-like particles by incubating purified monomeric influenza A/NP_∆14_ with a short synthetic single-stranded ^5′^P-(UC)_9_-FAM^3’^ RNA (18-mer) [22]. Of note, A/RNP-like particles were also obtained with different lengths of poly-UC RNA ranging from 12 to 18 nucleotides, however the double-stranded antiparallel helical assembly that is the most similar to A/vRNPs extracted from viruses was only resolved with the 18-mer RNA. To assess the ability of B/NP_WT_, B/NP_∆64_ and B/NP_∆68_ to form RNP-like particles, an incubation with a 12- and 24-mer RNA was set up overnight and observed by NS-EM. Inspection of the micrographs confirmed B/NP_WT_ inability to form helical filaments in any of these conditions as previously established by Labaronne *et al* [23], contrary to B/NP_∆64_ and B/NP_∆68_ for which RNP-like particles were observed with both RNA probes (Fig 2A). To determine the ideal conditions to assemble helical nucleocapsids using B/NP_∆64_ and B/NP_∆68_, we probed further poly-UC RNA with increasing lengths comprised of between 12 and 24 nucleotides (S2 Fig). All the RNA tested enabled the formation of helical filaments as well as smaller irregular assemblies resembling open and closed rings.

B/RNP-like particles formation was only observed with truncated NP, however the native vRNP are assembled with the full-length protein. To compare the morphology of B/RNP-like particles with native B/vRNP, we produced a recombinant B/Brisbane/60/2008 influenza virus expressing a PB2 protein fused to a strep-tag epitope at the C-terminus. Of note, the NPs from B/Brisbane/60/2008 and of the B/Memphis/13/2003 strains share 98,5% of sequence identity. The resulting PB2-Strep virus was efficiently rescued by reverse genetics and remained genetically stable upon subsequent amplification. After lysis of the virus, the Strep-tagged B/vRNP were purified by strep-tactin affinity chromatography [28] and visualized by NS-EM (Fig 2B). The morphology of native B/vRNP observed by NS-EM appears identical to that of RNP-like particles reconstituted *in vitro* with recombinant truncated B/NP and synthetic RNA, displaying characteristic major and minor groove features observed with native vRNP extracted from influenza A and D viruses [17–19,25].

This result indicates that *in vitro* reconstituted RNP-like particles are a relevant model to study the RNA encapsidation by nucleoproteins and the resulting helical architecture. However, due to the challenges in obtaining high-resolution structures for native vRNPs, some intrinsic structural differences may still exist. Here, B/NP_∆64_ and B/NP_∆68_ incubated with the 24-mer RNA form the most abundant nucleocapsid-like structures with minimal smaller particles, therefore yielding the least heterogeneous and most suitable samples for structural analysis by cryo-EM.

### Cryo-EM structure of B/RNP-like particles

**Fig 3:**
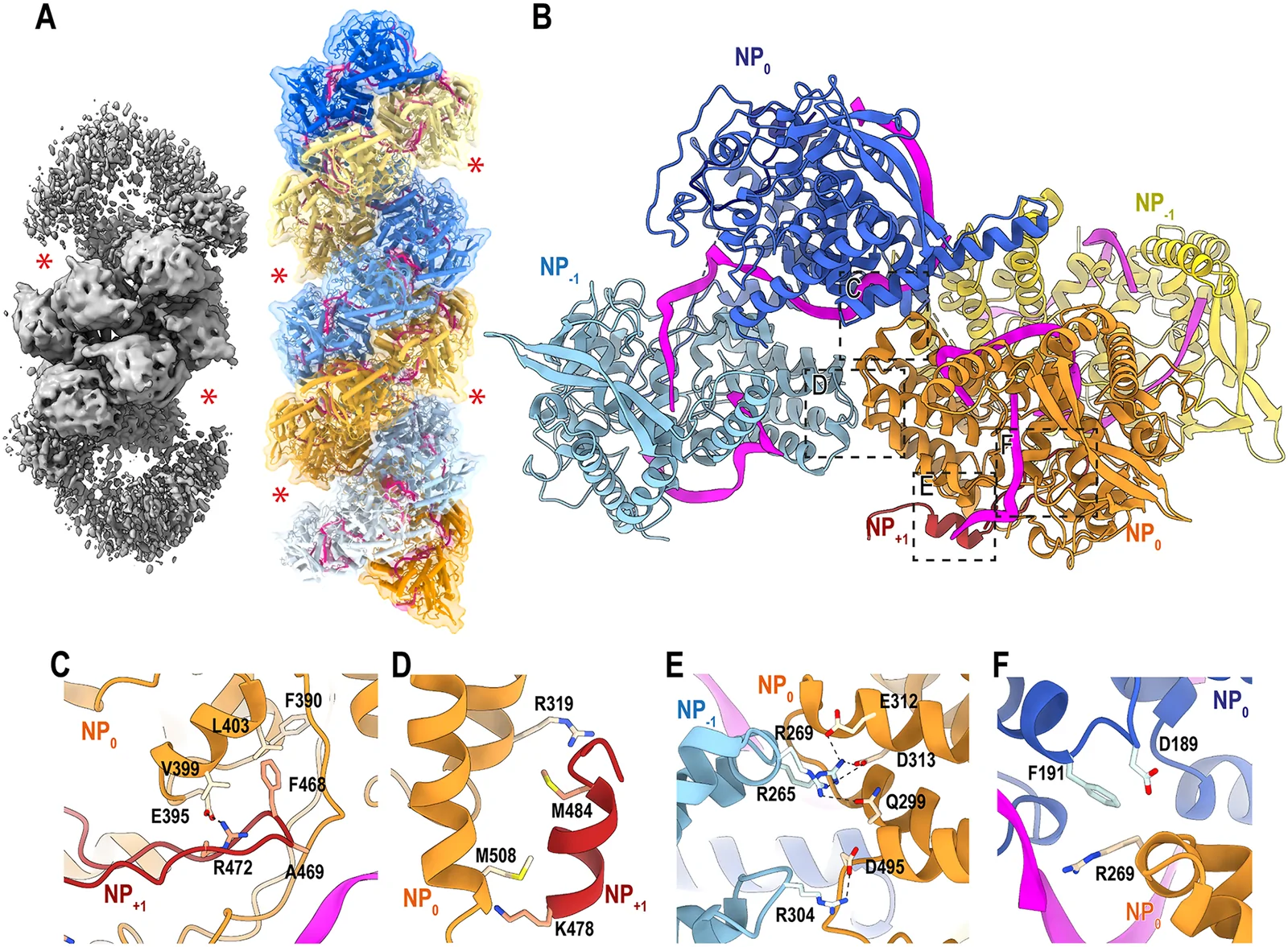
Cryo-EM structure of B/NP_∆68_ RNP-like particles. **A.** Left, cryo-EM 3D reconstruction of B/NP_∆68_ RNP-like particle at 6 Å resolution. Right, fitting of B/NP_∆68_ atomic model in the helically symmetrized cryo-EM structure. The antiparallel helical structure displays two turns. Each strand is colored with a sky-blue-to-blue or light-yellow-to-orange color gradient to indicate the positive polarity of each strand (light toward dark color). Red asterisks indicate the major grooves. **B.** Atomic model built from the focused refinement cryo-EM map encompassing 2-2 NPs. Consecutive NPs from the Strand-1 are colored in gradient of blue, while NPs on the Strand-2 appear in gradient of orange. The RNA is shown as a pink ribbon. **C-F.** Close-ups on residues involved in NP-NP and NP-RNA interactions. NP protomers are displayed in a cartoon representation, with colors identical to **B**. Dotted lines represent putative hydrogen bonds.

Cryo-EM data sets of B/NP_∆64_ or B/NP_∆68_ incubated with the 24-mer RNA were acquired using a Talos Glacios 200 kV transmission electron microscope (TEM), 2053 movies with a 1.145 Å pixel size and 5362 movies with a 0.93 Å pixel size were collected, respectively (S1 Table, S3 and S4 Figs). Consistent with the published A/RNP-like and D/vRNP structures [24,25], the analysis of both data sets resulted in the 3D reconstruction of a right-handed antiparallel double-stranded helical assembly with a minor and major groove (Fig 3A). Local refinement was performed on 2–2 NPs, consisting of two consecutive NP protomers face-to-face with two NP protomers on the opposing strand. This strategy improved the cryo-EM map local resolution to 5.3 Å and 4.1 Å for B/NP_∆64_ or B/NP_∆68_ RNP-like structures, respectively. Although the resolution differs, the two maps display the exact same 3D conformation (S5 Fig)**,** suggesting the 4 residues difference between the truncated B/NP constructions does not impact the RNP-like assembly overall. An atomic model was built using the best map starting from the B/NP crystal structure (PDB code: 3TJ0) (Fig 3A right and 3B). In the B/NP crystal structure, residues 127–146 are not resolved, however in the B/RNP-like structure, they clearly form an alpha-helix folding and hovering over the RNA. The density corresponding to a single stranded RNA is distinctly visible, yet the resolution varies across the NP interface binding and does not permit *de novo* model building. Thus, RNA from the A/RNP-like structure was used as a guide to manually place the RNA in this model. Despite these limitations, the resulting structure reveals the RNA pathway, as well as the inter- and intra-strands interactions with the helix.

### Helical architecture of B/RNP-like particles

Along one helical strand, direct interactions between two consecutive NP occur primarily via the oligomerization loop (OL) and the RNA situated at the NP-NP interface. The OL of NP_+1_, comprised of residues G458-M484, channels through the body of the neighboring NP_0_ protomer (Fig 3B-3D). Interpretation of the map with PDBePISA [29] suggests that the OL is stabilized mainly by a salt bridge between R472^NP+1^ and E395^NP0^ and hydrophobic interactions between F468^NP+1^ and F390^NP0^, V399^NP0^ and L403^NP0^ (Figs 3C and S1). The short alpha-helix, comprised of residues K478 to M484, at the end of the OL also interacts with the NP_0_ protomer head domain, possibly through electrostatic interactions between the residues K478^NP+1^ – M508^NP0^ and M484^NP+1^ – R319^NP0^ (Fig 3D). Given the resolution of the map, these interpretations should be considered with caution; nonetheless, all these interactions were previously observed in the tetrameric B/NP crystal structure and some were proven mandatory for the oligomerization of the protein [26]. Contrary to the A/RNP-like structure in which the S413 residue interacts with the RNA base through the hydroxyl group of the side chain, the equivalent residue A469 located at the very tip of the OL comes to close vicinity to the RNA without evidence of direct interaction.

**Fig 4:**
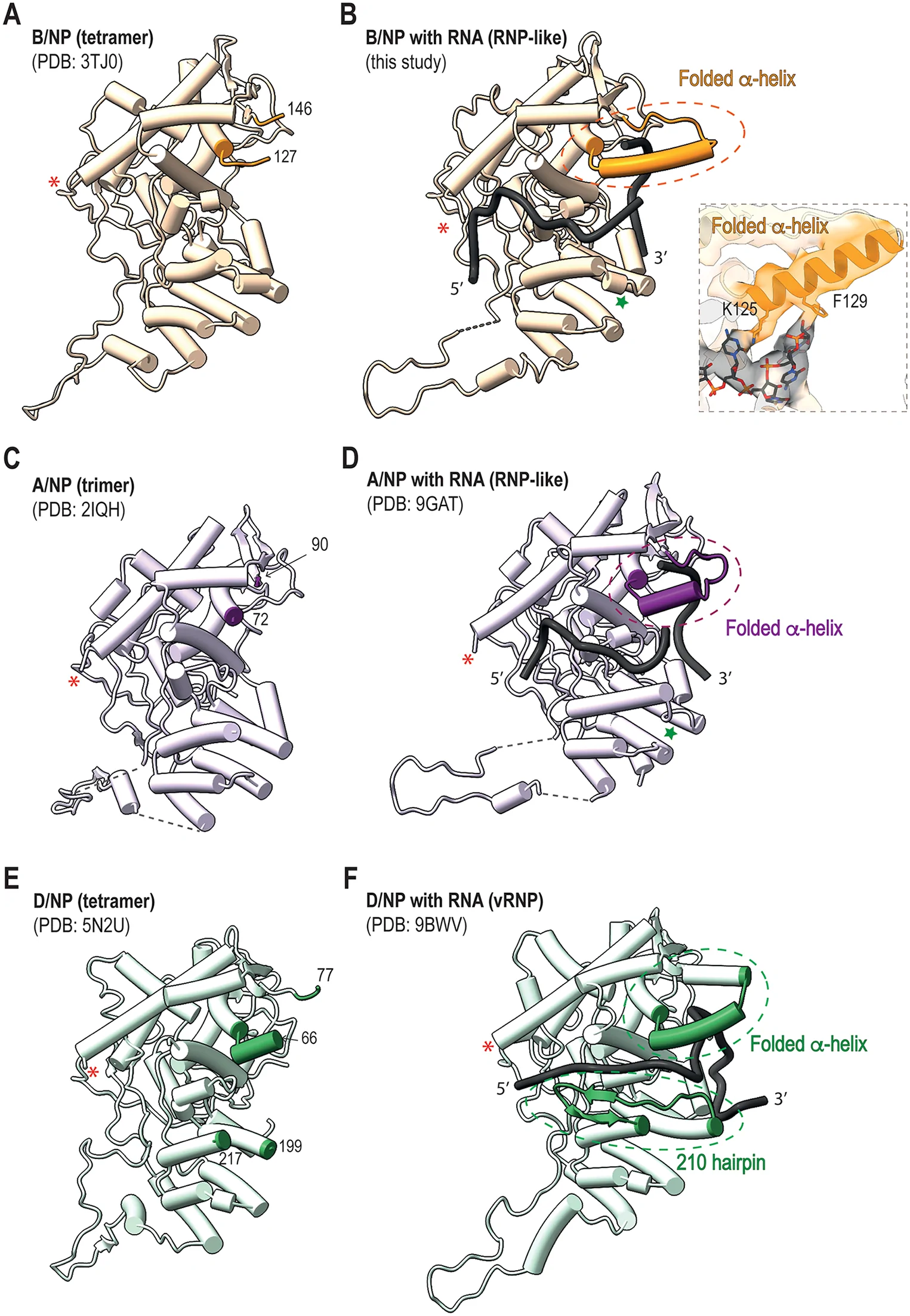
Conformational changes induced by RNA binding. **A, C, E.** Cartoon representations, with cylindric α -helices, of crystal structures of a single protomer of B/NP, A/NP and D/NP from trimeric or tetrameric oligomers in the absence of RNA. The N-terminal is marked with a red asterisk, unfolded loops 127-146 in B/NP, 72-90 in A/NP and 66-77, 199-217 in D/NP are highlighted in orange, purple and green, respectively. **B, D, F.** Cartoon representations, with cylindric α-helices, of single protomers of B/NP, A/NP and D/NP from RNP-like or vRNP cryo-EM structures. RNA is shown as a dark grey line, and N-termini are marked with a red asterisk. Newly folded α-helices and ß-strands are highlighted with darker color and a dotted line. The green stars (**B, D**) indicate the short loops in A/NP and B/NP while in D/NP the corresponding longer loop folds into the 210 hairpin **(F)**.

The RNA strongly contributes to the intra-strand cohesion by bridging two consecutive NP. In our B/RNP-like cryo-EM map, we observe a discontinuous density attributable to the RNA in both helical strands. The RNA can be observed weaving across the central basic groove of NP, and also at the interface with the neighboring NP, following the same pathway as observed in the D/vRNP and A/RNP-like structures. Residues 127–146, folded into an alpha-helix in our cryo-EM structure, which close around the RNA, while they are unresolved in the crystal structure without RNA bound [26]. Specifically, K125 points directly toward the RNA, and F129 appears to associate with the RNA by stacking interactions (Fig 4B). This observation suggests that RNA binding enables this otherwise flexible loop to fold.

PDBePISA analysis of the antiparallel double helical assembly identified two additional interfaces stabilized via inter-strand interactions. The first one, situated between the head domains of opposite protomers, possibly involves the arginines 265, 269 and 304 interacting via putative hydrogen bonds with Q299, E312, D313 and D495 (Fig 3E). The second interface is located between the head domain and the body domain of interfacing protomers, where R269 seems to be interacting with F191, and D189 and points directly toward the RNA of the opposite NP protomer (Figs 3F and S1). However, greater emphasis should be placed on the interface area rather than on individual residues due to the resolution of the map. In the A/RNP-like structure, R204 and R208 – corresponding to R265 and R269 in B/NP – are also key residues involved in inter-strand interactions, indicating their importance to the helical architecture.

Comparing the RNA pathway along the RNP-like helical Strand-1 and -2, we observe a continuous density within Strand-1 that fits 19 nucleotides, while the RNA density of Strand-2 appears discontinuous on one side of NP (S6 Fig). Although NP protomers share the same spatial distribution – or helical parameters – on Strand-1 and -2, NPs located on the Strand-1 are slightly tilted inward compared to NPs on Strand-2. Consequently, the RNA is more exposed to the solvent in Strand-2, which may explain the density discontinuity.

## Discussion

In this study, we characterized the NP-RNA complex of an influenza B virus using recombinant B/NP protein and a short synthetic RNA. Following a strategy inspired by our previous work with A/NP [22,24], the full-length B/NP and two N-terminal tail truncations (B/NP_WT_, B/NP_∆64_ and B/NP_∆68_, respectively) were generated, and their ability to bind RNA was tested. All three constructs were purified as monomers and bound RNA with similar affinity. However, the formation of RNP-like particles was only achieved with the truncated proteins B/NP_∆64_ and B/NP_∆68_, and their structures were solved by cryo-EM single particle analysis at 5.3 and 4.1 Å resolution respectively. Since both samples were prepared in the same conditions, the difference in resolution comes most likely from the amount of data collected, 2053 movies for B/NP_∆64_ versus 5362 movies for B/NP_∆68_, and the camera performance (movies were recorded with a K2 summit camera with B/NP_∆64_ and a falcon4i camera with B/NP_∆68_), resulting in more particles of better quality used for the final 3D reconstruction. Nonetheless, the overall architecture of both structures is identical and NP residues 70–548 are visible in both maps.

**Fig 5:**
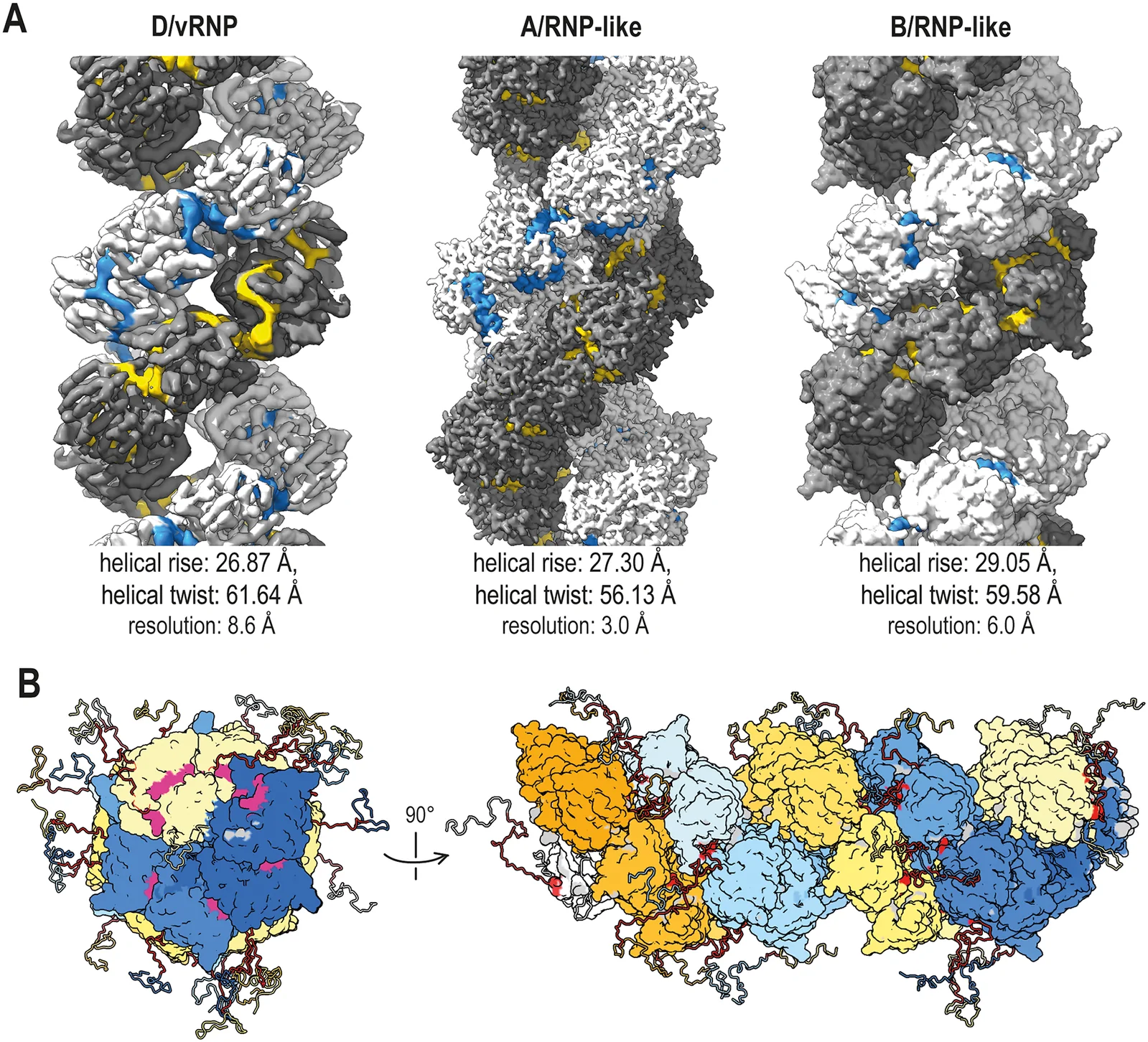
A. Comparison of D/vRNP (EMDB: 44980), A/RNP-like (EMDB: 51188) and B/RNP-like structures. Each double stranded antiparallel helical map is displayed with one strand colored in white (NP) and blue (RNA), and the other strand in grey (NP) and yellow (RNA). The applied helical parameters and resolutions are indicated below each structure. **B.** Proposed model of the location of the B/NP disordered N-terminal tail in the helical RNP architecture. Models were generated with the RANCH program and fitted into our experimental cryo-EM structure. Views are from the top (**left**) or side (**right**) of the helical filament shown as a surface. NPs on Strand-1 are colored in a blue gradient and NPs on Strand-2 are colored in an orange gradient. RNA is colored in grey. The modeled disordered tails are shown as cartoons and residues of the NLS are colored in red.

We characterized the B/RNP-like structure assembled in an antiparallel double stranded helical conformation. It adopts the same architecture that was previously described with the *in vitro* reconstituted A/RNP-like structure, and with the D/vRNP NS segment extracted from cells. The right hand-ness is conserved across all 3 structures as well as the RNA pathway and the typical major and minor groove features (Fig 5A). Interestingly, the native D/vRNP doesn’t display any inter-strand interaction, contrary to the reconstituted A/- and B/RNP-like structures, where NP-NP inter-strand direct interactions contribute to the helical assembly stabilization. This could be explained by the presence of the 50 residues long disordered tail at the C-terminal end of D/NP that is unresolved in the cryo-EM structure yet generates some spatial distancing between the two strands and flexibility in the vRNP. As a consequence of the close inter-strand interaction in A/- and B/RNP-like structures, the RNA on Strand-1 is largely buried and hidden from the solvent, while in the native D/vRNP structure, the RNA appears more accessible regardless of the strand, and the helical architecture altogether loser. This suggests that native vRNP are more flexible, probably to accommodate the access of FluPol to the viral RNA and allow polymerase processivity, and to allow protruding RNA regions for vRNP packaging.

Another common feature shared by these 3 structures is the folding of the loop located between the alpha helix α2 and beta strand β1 upon RNA binding (residues 72–90, 124–146 and 66–77, in A-, B- and D-NP respectively), allowing positively charged residues to lock the RNA in the central binding groove (Fig 4). The D/NP has an additional loop, residues 199–217, that folds into a small antiparallel β-sheet in the context of the vRNP, and that is not present in A/NP and B/NP (Figs 4 and S1). This second folded loop appears to be tightly holding the RNA in the basic groove, contrary to A/- and B/RNP-like structures in which the RNA electron density is less defined and discontinuous in the same region, possibly due to the empty space in lieu of the small antiparallel β-sheet. Altogether, this study extends the previously established principles for NP-RNA interaction and helical assembly [22,24,25] to B/RNP-like particles.

Among the *Orthomyxoviridae*, vRNP flexibility has limited the obtention of structures describing the interaction of the nucleoprotein with genomic RNA [17,18,30]. Alternative strategies using smaller entities such as N-RNA rings, mini-RNP or RNP-like particles enabled the observation of RNA bound to nucleoproteins. Indeed, the Tilapia Lake virus N-RNA ring [31], the influenza A virus mini-RNP [21] and influenza A virus RNP-like [24] structures enabled the modeling of 12, 10 and 18 nt per protomer, respectively, with evidence of additional space to fit more nt. In this study, a 24-nt synthetic RNA was used to assemble the influenza B virus RNP-like filaments. Although the EM density corresponding to the RNA is weakly defined, 19-nt were modeled per NP protomers in both helical strands, with a gap in density between consecutive RNA molecules that could fit 2–3 additional nucleotides. This is consistent with a previous analysis reporting that in the context of the vRNP, the measured ratio between nucleotides and NP is 24 nt/NP [20].

With this work, we demonstrate that the 70 amino-acid disordered N-terminal tail of B/NP does not impact RNA binding *in vitro*, nor the oligomeric state of purified B/NP constructions, however, helical particles were only obtained using the truncated constructions in the RNP-like particles reconstitution assay *in vitro*. Within the native vRNP, the helical architecture is assembled with the full-length NP, which suggests the disordered tail may introduce steric hindrances that interfere with efficient assembly *in vitro*. The absence of additional viral or host factors present in the native environment might also affect the kinetic and dynamic of the helical particle formation and contribute to this discrepancy *in vitro.* In the context of viral infection, the N-terminal tail contains the Nuclear Localization Signal (NLS) [32,33] that binds to human importin-α [27], yet the role of such an extended N-terminal region, compared to 20 amino-acids for A/NP, remains unknown. In D/NP, the NLS is located on a 50 amino-acid disordered tail at the C-terminal end of the protein, and is not visible in the D/vRNP cryo-EM structure. Altogether, our data shows that the disordered N-tail does not participate to the helical stabilizing interactions within the RNP-like structure; nonetheless, the possibility that the tail plays a non-structural role in the proper helical assembly of vRNPs cannot be excluded.

When modeling the intrinsically disordered B/NP N-terminal tail upon the cryo-EM structure of the folded domain with an ensemble optimization method (EOM) [34,35], the different conformers illustrate the plasticity of the N-terminal tail (S7 Fig). In the context of the RNP-like structure, the conformers appear to partially shelter the helical nucleocapsid from the solvent while exposing the NLS (Fig 5B). In the Wuhan spiny eel Influenza virus – a Wuhan Asiatic toad Influenza virus identified lately [36], the NP for the toad virus (toad/NP) has 30% identity with B/NP and possess a very long N-terminal tail (126 residues) of unknown function [37]. In measles virus, the disordered C-terminal tail of the nucleoprotein binds to the viral phosphoprotein, and modulates the polymerase activity [38,39]. In influenza B viruses, the role of the long disordered NP tail remains unclear beyond its function in nuclear localization, but it may participate in the recruitment of viral and/or cellular cofactors.

## Materials and methods

### Cloning and plasmid preparation

The full-length NP, called B/NP_WT_, (B/Memphis/13/03) was cloned in the pETM11 vector (EMBL) to be expressed as N-terminal His-tag protein (as described in [27]). The N-terminal truncations B/NP 65–562 and B/NP 69–562 (called B/NP_∆64_ and B/NP_∆68_ respectively) were cloned in pET22b (EMBL) by PCR amplification using 5′ end Nde1 and 3′ end Xho1 to be expressed as C-terminally His-tagged proteins.

The eight bidirectional pDP2002-Bris plasmids used to generate B/Brisbane/60/2008 viruses by reverse genetics were kindly provided by Pr. D. Perez (College of Veterinary Medicine, University of Georgia) [40]. To introduce a Strep-tag II at the C-terminus of the PB2, the strep-tag sequence was introduced in place of the 2A-mCherry of the pDP2002-Bris-PB2-2A-mCherry (kindly provided by Pr S. Munier) carrying a duplication of the last 153 nucleotides of the PB2 open reading frame and silent mutations in the corresponding region actually encoding the PB2 protein [41].

### Cells

Human embryonic kidney 293T (HEK293T) (ATCC CRL-3216) were grown in Dulbecco’s modified Eagle’s medium (DMEM) supplemented with 10% fetal bovine serum (FBS) and 1% penicillin-streptomycin. Madin-Darby canine kidney (MDCK) (provided by the National Reference Center for Respiratory Infections, Institut Pasteur) were grown in modified Eagle’s medium (MEM) supplemented with 5% FBS and 1% penicillin-streptomycin.

### Recombinant protein expression and purification

The expression and purification of B/NP_WT_ B/NP_∆64_ and B/NP_∆68_ was completed as previously described by [27]. Briefly, each plasmid construct was transformed into *Escherichia coli* BL21 RIL (Life Technologies) and expression was induced with 0.3 mM isopropyl-β-D-thiogalactopyranoside (IPTG, Neo Biotech) overnight at 18 °C. Bacterial pellets were resuspended and sonicated in lysis buffer (50 mM Hepes pH 7.5, 300 mM NaCl, 1 M 3-(1-pyridinio)-1-propanesulfonate (NDSB), 5 mM β-mercaptoethanol (βME) and cOmplete EDTA-free Inhibitor Cocktail (Roche)). Proteins were first purified by Ni^2+^ affinity chromatography using gravity (Ni-NTA, Qiagen) and eluted in 20 mM Hepes pH 7.5, 300 mM NaCl, 500 mM imidazole, 5 mM βME. Followed by a HiTrap Heparin HP 5 mL column (Cytiva) eluted with a NaCl gradient. Heparin elution fractions were dialyzed overnight at room temperature against 20 mM Hepes pH 7.5, 50 mM NaCl, 5 mM βME. Proteins were then concentrated on 10 KDCO Amicon filters (Millipore) and further purified by size exclusion chromatography using a Superdex75 10/300 GL column (Cytiva) equilibrated with 20 mM Hepes pH 7.5, 50 mM NaCl, 5 mM βME.

### Fluorescence anisotropy

Serial dilutions of B/NP_WT_ or B/NP_∆64_ were performed in 20 mM Hepes pH 7.5, 50 mM NaCl, 5 mM βME buffer, and distributed in black 384-well plates (Greiner Bio-One). Synthetic single-stranded poly-UC RNA molecules with a fluorescein (FAM) attached at the 3’end were diluted to 5 nM in a final volume of 60 μl. RNA was added last to NP dilutions, and the mixtures were incubated for 5 h before anisotropy measurements. The experiments were performed in triplicate on a Clariostar microplate reader (BMG Labtech), using 480 nm wavelength for excitation and emission was recorded at 520 nm. After removing anisotropy values corresponding to RNA-FAM alone, the data were plotted with GraphPad Prism and dissociation constants (Kd) were calculated using the single binding site with Hill slope (h) function.

### RNP-like particles assembly

The RNP-like filaments were prepared as previously described [24]. Briefly, 100 µM B/NP proteins and 100 µM RNA molecules were incubated overnight in 20 mM HEPES pH 7.5, 150 mM NaCl, 5 mM βME. The UC-repeated sequence was used for its inability to form secondary structures. Several RNA lengths (12-, 16-, 20- and 24-mer) were tested for RNP-like particles assembly and visualized by negative stain electron microscopy (NS-EM).

### B/vRNP production and purification

For production of recombinant influenza B virus (IBV), a co-culture of HEK293T and MDCK cells (4 × 10^5^ and 3 × 10^5^ cells, respectively, seeded in a 6-well plate) were transfected with the pDP2002-Bris plasmids, as described previously [40]. Virus supernatant was titrated on MDCK cells by plaque assay [42] with plaque diameters measured with Fiji [43]. Virus was then amplified at an MOI of 0.0001 on MDCK cells in DMEM containing 1 μg/mL of TPCK-treated trypsin for 3 days at 35°C. Supernatants were harvested and clarified by centrifugation for 5 min at 2,500 g before storage at -80°C.

Virus was harvested from infected cell supernatants by centrifugation at 15,000 rpm with a JA 25.50 rotor for 45 min at 4 °C. The pellet was resuspended in lysis buffer (50 mM HEPES (pH 8), 1% (w/v) Triton X-100, 200 mM NaCl, 5 mM MgCl_2_, 5% (v/v) glycerol, 2 mM TCEP, murine RNase inhibitor (40 U/ml) (NEB), lysolethicin 1 mg/ml and cOmplete EDTA-free Inhibitor Cocktail (Roche)) and incubated for 2 h at 4 °C with gentle rocking. B/vRNP were purified on Strep-Tactin Sepharose (IBA) by the batch method with centrifugation steps of 500 x g for 30 sec at 4 °C. Subsequently, protein was eluted in 20 mM of HEPES pH 8, 150 mM KCl, 1 mM MgCl_2_, 2 mM TCEP and 50 mM biotin and kept at 4 °C.

### Negative stain Electron Microscopy

A 4-µL drop of purified protein, freshly assembled RNP-like particles or freshly purified B/vRNPs diluted to 0.5-0.7 µM was applied onto glow discharged Formvar/Carbon Support grid (Quantifoil) and incubated for 1 min. The sample solution was absorbed by gentle side blotting and the grid was immediately stained with 2% (w/v) Uranyl acetate for 1 min. The grid was then blotted dry at room temperature and imaged on a Tecnai F20 electron microscope (FEI Tecnai, Hillsboro, OR, USA) equipped with an FEI Ceta camera and operating at 200 kV at a nominal magnification of ×55000.

### Cryo-grid preparation and data collection

A 4-µL drop of sample diluted to 20 µM was applied onto an UltrAufoil 1.2/1.3 300 mesh grid (Quantifoil) previously glow-discharged during 45 s at 25 mA (EMS glow discharger). The grid was incubated 10 s at 20 °C under 100% humidity using a Mark IV Vitrobot (Thermo Fisher Scientific), before blotting 5 s using force 0, and plunge-frozen in liquid ethane. For the B/NP_∆64_ sample, automated data collection was performed on a TEM Glacios FEG microscope (Thermo Fisher Scientific) operated at 200 kV equipped with a K2 summit direct electron detector camera (Gatan). Coma and astigmatism correction were performed on a carbon grid, and 2,053 movies of 50 frames were collected using SerialEM [44], with a dose rate of 1 e^-^/Å^2^ per frame. Movies were recorded at a nominal x36,000 magnification giving a 1.145 Å pixel size with a defocus ranging from -0.6 to -2.0 µm. For the B/NP_∆68_ sample, the TEM Glacios FEG microscope had been upgraded with a Falcon4i camera (Thermo Fisher Scientific). 5,362 movies of 45 frames were automatically recorded using EPU (Thermo Fisher Scientific) with a dose rate of 0.85 e^-^/Å^2^. Movies were recorded with a defocus ranging from -0.6 to -2.0 µm, at a nominal x36,000 magnification giving a 0.93 Å pixel size.

### Cryo-EM image processing and model building

With the B/NP_∆64_ sample (S3 Fig), movies were imported in Relion 4.0 [45] and drift correction was performed with Relion’s MotionCorr2 implementation using 5x5 patch on all frames. Aligned and dose weighted micrographs were then imported into CryoSPARC v4.7.0 [46] to determine contrast transfer function (CTF) parameters using patch CTF estimation. Realigned micrographs were manually curated to exclude those of low quality regarding CTF fit, ice thickness and out of range defocus. Initial particle picking was performed with filament tracer by using a diameter of 150 Å, minimum length of 300 Å and filament segmentation every 50 Å, followed by several rounds of 2D classification. The resulting 2D classes were used as templates to select 1,154,916 particles, extracted into 400 x 400 pixels^2^ boxes. After three rounds of 2D classification, an Ab Initio model was generated with 159,516 particles and further refined with a central mask covering 30% of the total box. The helical parameters of the resulting map were assessed using HI3D in real space [47], estimating a rise of 26.27 Å, and a 60.31° twist. Based on the helical parameters, particles having a separation distance under 90 Å (corresponding roughly to three times the rise parameter) were removed to avoid signal duplication during further masked processing. Particles were symmetry expanded using the estimated rise and twist with an order of three, then subtracted in Relion 4.0 using a mask encompassing four NP protomers (two NP on one helical strand and two facing NP on the opposite strand). After further local refinement, the estimated resolution indicates 5.3 Å.

With the B/NP_∆68_ data set (S4 Fig), movies were imported in CryoSPARC v4.7.0 and processed with patch motion correction and patch CTF estimation. 4,177,714 particles were selected on curated micrographs by template picking, using the structure determined above and a separation distance close to the rise (30 Å). Particles extracted into 400 x 400 pixels^2^ boxes were subjected to several rounds of 2D classification. The Ab-Initio model was generated with 323,605 particles and further refined with a central mask covering 30% of the total box. Homogeneous refinement was performed with a central mask covering 30% of the total box. Particles under 75 Å distance were removed to avoid signal duplication. Precise helical parameters were calculated using HI3D in real space, giving a rise of 29.05 Å and a 59.58 ° twist, and used for symmetry expansion using an order of two. After local refinement with a mask encompassing four NP protomers, the estimated resolution indicates 4.1 Å. Local resolution of each final map was estimated using the CryoSPARC suite and the B/NP_∆68_ map was post processed with the EMReady algorithm [48].

The B/NP_∆68_ atomic model was manually built in COOT [49] and guided with the B/NP atomic model (PDB: 3TJ0) rigid body fitted in the EMready modified map. Although the resolution does not allow high confidence to model the RNA molecule, the RNA from A/RNP-like structure (PDB: 9GAT) was utilized and manually adjusted to fit the EM density. The model was then refined using phenix real-space refinement [50] against the unmodified EM map. Atomic model validation was performed with MolProbity [51] and the PDB validation server. A helical atomic model of the RNP-like particle was generated by symmetry using the 2–2 NP atomic model built in the final EM map. All figures were prepared with ChimeraX version 1.9 [52].

### Modeling of NP N-ter tail in the context of the RNP helical assembly

NP N-ter tail was built onto the atomic model of B/NP derived from the B/RNP-like particle EM density. Disordered N-ter tails conformers were randomly generated based upon sequence using an ensemble optimization method (EOM) with RANCH (RANdom CHains) algorithm from the EOM 3.0 suite [34,35] while maintaining structural constrains on the NP core domain.

## Supporting information

S1 Fig Sequence alignment of B/NP (B/Memphis/13/2003), A/NP (A/WSN/1933/H1N1) and D/NP (D/swine/Oklahoma/1334/2011). Residues are colored according to conservation, with identical residues highlighted in red, equivalent highlighted white with red text and non-conserved not colored. The NLS sequence are underlined in orange for each NP. Secondary structures appear above the sequences in black, alpha helices and beta hairpin that specifically fold upon RNA binding are colored in green. Blue triangles indicate conserved residues involved in NP-NP or NP-RNA interaction.
(TIF)

S2 Fig RNP-like formation observed by electron microscopy with negative staining. Comparison of RNA of different length (12-, 16-, 20- and 24-mer) incubated with either B/NP_∆64_ or B/NP_∆68_. Helical filaments were observed in all conditions, with a higher proportion in the presence of the 24-mer RNA. Scale bars represent 100 nm.
(TIF)

S3 Fig Cryo-EM image analysis workflow for the B/NP_∆64_–24-mer RNP-like complex. Data processing steps were performed in Relion 4.0 [45] and CryoSPARC v4.7.0 [46]. Details in the Materials and Methods section. White scale bar represents 100 nm.
(TIF)

S4 Fig Cryo-EM image analysis workflow for the B/NP_∆68_–24-mer RNP-like complex. Data processing steps were performed in CryoSPARC v4.7.0 [46]. Details in the Materials and Methods section. White scale bar represents 100 nm.
(TIF)

S5 Fig Comparison of the cryo-EM maps obtained with B/NP_∆64_–24-mer and B/NP_∆68_–24-mer displayed with the orientation. **A.** Cryo-EM maps of the B/RNP-like obtained with B/NP_∆64_–24-mer. Top: 3D reconstruction at 6.1 Å resolution after helical alignment and symmetry expansion. Strand-1 is colored in gradient of green, Strand-2 appears in gradient of purple and RNA in red. Bottom: 3D reconstruction at 5.3 Å resolution after local refinement focused on 4 central NPs. For clarity, only NP_0_-NP_-1_ of Strand-2 are displayed. **B.** Cryo-EM maps of the B/RNP-like obtained with B/NP_∆68_–24-mer. Top: 3D reconstruction at 6.2 Å resolution after helical alignment and symmetry expansion. Strand-1 is colored in gradient of blue, Strand-2 appears in gradient of orange and RNA in red. Bottom: 3D reconstruction at 4.1 Å resolution after local refinement focused on 4 central NPs. For clarity, only NP_0_-NP_-1_ of Strand-2 are displayed. Illustrations were prepared with ChimeraX version 1.9 [52].
(TIF)

S6 Fig Comparison of the RNA EM density locally refined between Strand-1 and Strand-2. Both EM maps are displayed at the same 3.0 threshold, the density for NP is colored in blue in Strand-1 and in yellow in Strand-2. RNA density colored in pink is continuous across the NP central binding groove in Strand-1 and discontinuous in Strand-2. Illustrations were prepared with ChimeraX version 1.9 [52].
(TIF)

S7 Fig Conformational ensemble (24 conformers shown out of 40) of the full-length B/NP calculated from the atomic model of NP_0_ in Strand-1 and the RANCH program to generate random models of the disordered N-terminal tail. The NP core that remains unchanged is displayed as a blue surface. The multiple N-terminal tail conformations are shown in cartoon colored in grey and in red to locate the NLS. The NP Cter and a few Nter ends are labeled. Illustration was prepared with ChimeraX version 1.9 [52].
(TIF)

S1 Table Cryo-EM data collection parameters, refinement and validation statistics. (XLSX)
