# Correction: Nucleocapsid-like cryo-EM structure of influenza B virus

**DOI:** 10.1371/journal.ppat.1014784

**Published:** 2026-11-12

**Authors:** Marie Thirion, Alice J. Stelfox, Florian Chenavier, Héléna Chomat, Catherine Isel, Lily-Lorette Freslon, Eleftherios Zarkadas, Rob W. H. Ruigrok, Nadia Naffakh, Thibaut Crépin, Allison Ballandras-Colas

The following information is missing from the Acknowledgement section: This work used the platforms of the Grenoble Instruct-ERIC center (ISBG; UAR 3518 CNRSCEA-UGA-EMBL) within the Grenoble Partnership for Structural Biology (PSB), supported by FRISBI (ANR-10-INBS0005-02) and GRAL, financed within the University Grenoble Alpes graduate school (Ecoles Universitaires de Recherche) CBH-EUR-GS (ANR-17-EURE-0003). The IBS-ISBG EM facility is supported by the Auvergne-Rhône-Alpes Region, the Fondation Recherche Medicale (FRM), the fonds FEDER and the GIS-Infrastructures en Biologie Santé et Agronomie (IBISA).
