# Supplementary material for: Nucleocapsid-like cryo-EM structure of influenza B virus
Source: PLoS Pathog. 2026 Aug 10;22(8):e1014449. doi: 10.1371/journal.ppat.1014449 (PMC13497295; doi:10.1371/journal.ppat.1014449)
Supplement: Supplementary file 1 [file ppat.1014449.s010.docx]

We thank the reviewers for their attentive reading of our work and constructive remarks, which has helped us to improve the manuscript. Specific points are discussed directly in the text below (reviewers in black, our response in blue for clarity).

**Major Issues**

Reviewer #1:

1. The main structural conclusion that influenza B RNP-like particles adopt a right-handed antiparallel helical organization similar to influenza A and D vRNPs is consistent with recent studies on influenza A and D virus nucleocapsids. However, the discussion should better acknowledge that the major principles of NP-RNA organization and helical assembly were already established in Chenavier et al., Sci Adv 2023; Chenavier et al., NAR 2025; Peng et al., Science 2025.

These three papers are well cited throughout all sections of the manuscript, nonetheless, we modified the sentence line 285-286 of the discussion: from “These observations show that the vRNPs from Influenza virus type A, B and D share the same right-handed antiparallel helical conformation” to “Altogether, this study extends the previously established principles for NP-RNA interaction and helical assembly (22,24,25) to B/RNP-like particles.” With references 22,24 and 25 being: Chenavier et al., Sci Adv 2023; Chenavier et al., NAR 2025; Peng et al., Science 2025.

2. The conclusion that the B/NP N-terminal tail “does not participate to the helical architecture” should be softened. The data convincingly show that truncated proteins can assemble into helical particles and that the tail is not required for RNA binding in vitro or for the formation of RNP-like architecture under the tested conditions. However, it cannot be ruled out that the tail could plays non architectural role in native vRNPs essential to its proper helical assembly. This is especially important given the proposed flexibility and potential regulatory functions discussed later in the manuscript. I suggest rephrasing this conclusion more cautiously throughout the manuscript.

We do agree that we cannot rule out that the tail plays a non-architectural role in native vRNP assembly into a proper helical filament. However, the tail is absent from the structures of native D/vRNP (however the disordered tail is positioned at the C-terminal end) and A and B/RNP-like particles suggesting that the tail is not required to structurally stabilize helical interactions. In order to clarify that point, we included the following modifications:

Line 29-32 (Abstract) we added “stabilization once the vRNPs are assembled” to the sentence: “Overall, our results suggest that vRNPs from Influenza virus type A, B and D share the same right-handed antiparallel helical conformation and that the B/NP N-terminal tail does not participate to the helical architecture **stabilization once the vRNPs are assembled”.**

Line 316-318 (Discussion) modified from “suggesting that it does not participate to the vRNP helical architecture” to “Altogether, our data shows that the disordered N-tail does not participate to the helical stabilizing interactions within the RNP-like structure; nonetheless, the possibility that the tail plays a non-structural role in the proper helical assembly of vRNPs cannot be excluded.”

3. The statement that the reconstituted particles “appear identical to vRNPs extracted from viruses” is overstated. The comparison with native B/vRNPs relies mainly on negative-stain EM morphology. While the resemblance is convincing at that level, the difficulties of obtaining high resolution structure from native vRNPS suggest some intrinsic structural differences. I recommend moderating the wording accordingly.

We moderated the text such that:

Line 27-29 (Abstract): “**Their morphology** appears identical to **native** vRNPs extracted from viruses **when observed by negative-satin electron microscopy**”

Line 166-173, we clarified by adding the following text in bold: “The **morphology of** native B/vRNP **observed by NS-EM** appears identical to that of RNP-like particles reconstituted *in vitro* with recombinant truncated B/NP and synthetic RNA, displaying characteristic major and minor groove features observed with native vRNP extracted from influenza A and D viruses (17–19,25).

This result indicates that *in vitro* reconstituted RNP-like particles are a relevant model to study the RNA encapsidation by nucleoproteins and the resulting helical architecture. **However, due to the challenges in obtaining high-resolution structures for native vRNPs, some intrinsic structural differences may still exist.**”

4. The authors appropriately acknowledge that the RNA density is not sufficiently resolved for de novo modelling. However, this limitation should also be extended to the interpretation of the protein model more generally, as the density displays relatively few well-resolved side chains. At this resolution, the map primarily supports rigid-body or flexible fitting rather than detailed atomic interpretation. Consequently, residue-level interactions should be interpreted cautiously. Nevertheless, I think the cryo-EM density remains sufficiently interpretable to support the main conclusions of the manuscript, especially since several of the interactions discussed (for example those described in Fig. 3C) are consistent with previously reported influenza NP crystal structures.

We agree that the protein model should be cautiously interpretated considering the map resolution.

The first section discussing residue-level interactions was nuanced by adding the following sentence line 212-213: **Given the resolution of the map, these interpretations should be considered with caution, nonetheless,** all these interactions were previously observed in the tetrameric B/NP crystal structure and some were proven mandatory for the oligomerization of the protein

The second section (line 231-239) was also toned down “The first one, situated between the head domains of opposite protomers, **possibly** involves the arginines 265, 269 and 304 interacting via **putative** hydrogen bonds with Q299, E312, D313 and D495 (Fig 3E). The second **interface** is located between the head domain and the body domain of interfacing protomers, where R269 seems to be interacting with F191, and D189 and points directly toward the RNA of the opposite NP protomer (Fig 3F and Suppl. Fig. 1). **However, greater emphasis should be placed on the interface area rather than on individual residues due to the resolution of the map**.**”**

5. The manuscript still lists placeholder accession numbers (“XXX, YYY, ZZZ”) in the Data Availability section and table 1. These must be completed before publication.

We’ve updated the manuscript with the accession numbers line 487-484: **“**Cryo-EM density maps of RNP-like particle resolved in this study and the associated atomic coordinates have been, respectively, deposited in the Electron Microscopy Data Bank (EMDB) and in the PDB under the following accession codes: **PDB_000031JO, EMD-58448, EMD-58449”**, as well as in Supplementary Table 1 in the supplementary information.

Reviewer #2: (No Response)

**Minor Issues**

Reviewer #1:

1. The authors may consider clarifying why the full-length B/NP fails to assemble into RNP-like filaments in vitro despite native vRNPs obviously containing full-length NP. A short discussion of possible steric or kinetic effects of the disordered tail during in vitro assembly would strengthen the interpretation.

We have expanded the discussion with the following text, line 303-311: “Within the native vRNP, the helical architecture is assembled with the full-length NP, which suggests the disordered tail may introduce steric hindrances that interfere with efficient assembly in vitro. The absence of additional viral or host factors present in the native environment might also affect the kinetic and dynamic of the helical particle formation and contribute to this discrepancy in vitro.”

2. Please ensure all supplementary figures and tables are appropriately referenced and described in the main text.

This point was carefully checked again, all supplementary figures and tables are correctly referenced.

Reviewer #2:

1. Throughout the text the use of B/RNP-like is an adjective without a noun – I suggest B/RNP-like particles, or B/RNP-like complexes.

We acknowledge that “RNP-like” was sometimes used as a noun, we fixed that issue directly in the text by adding “particles” when it was missing. Please refer directly to the manuscript.

2. Line 43. “extended N-terminal tail does not imper RNA binding ability and oligomeric state…” presumably the intention is ‘impair’ however while it doesn’t appear to have a material effect on RNA binding, it certainly does impact the oligomeric state.

We corrected the typo and modified the sentence to “the extended N-terminal tail does not **impair** RNA binding ability and **NP** oligomeric state in vitro.” to clarify that the oligomeric state of the purified NP protomers remains unchanged.

3. Figure 1C – the negative stain EM of the monomeric NP is difficult to interpret – presumably the white arrows indicate a random selection of the particles, but the image is of a field of particles, why use arrows? – are they pointing to a particular subset? The images are not very convincing concerning the homogeneity/monodispersity of the preparation as the particles are so small. Perhaps other methods may be more helpful, such as dynamic light-scattering or mass photometry (if they are available). The gel-filtration shows a reasonably tight-elution profile but would benefit from e.g. silver stained gel to show protein purity.

We are aware that visualization of the monomeric NP by negative staining electron microscopy is difficult to interpret, which is why we placed a few arrows to guide readers. These arrows do not point at a specific NP subset.

Regarding homogeneity/monodispersity, in our experience, and as published in Labaronne A. *et al.*, Viruses, 2016, NP mainly forms highly stable trimers and tetramers when purified in 300 mM NaCl. We were never able to assemble RNP-like particles starting from trimers/tetramers. To enable RNP-like complex formation, we need to maintain the protein in a primarily monomeric state, which is achieved with at 50 mM NaCl – however, removing NaCl altogether causes NP to precipitate. Moreover, SEC-MALS conducted on B/NPwt at 50 mM NaCl showed a homogeneous peak, consistent with a monomer of 67 kDa (Labaronne A. *et al.*, Viruses, 2016).

Altogether, while the preparation of NP_∆64_ and NP_∆68_ may not be perfectly monodispersed (as we did not investigate the truncated constructs with mass photometry or SEC-MALS), they appear mostly monomeric. Importantly, the truncated constructs display the same ‘imperfect’ dispersity under SEC and NS-EM as NP_wt_, and therefore, we believe we can fairly compare their ability to form RNP-like particles *in vitro* under these conditions.

4. Line 146 “Inspection of the micrographs confirmed B/NPWT inability to form helical filaments” why confirmed? Was this information already known?

Indeed this information was already known, we’ve added the reference supporting this statement: “Inspection of the micrographs confirmed B/NP_WT_ inability to form helical filaments in any of these conditions **as previously established by Labaronne et al (23)**”

5. Line 220 “… we observe a density inconsistent attributable to the RNA in both helical strands”. I am not sure what the intention is here – is the density inconsistent with being RNA? Or is the density weak or discontinuous?

The intention got lost in translation, we’ve changed inconsistent for discontinuous: “In our B/RNP-like cryoEM map, we observe a **discontinuous** density attributable to the RNA in both helical strands.”

6. Cryo-EM image processing methods. I note that the authors have used a more or less identical approach to determining the structure of the two N-terminally truncated NP-RNA complexes but implemented in Relion for one map and in CryoSPARC for the other. Is there a reason for this? Was the analysis performed by two operators with their own preferences, or did one software yield a better outcome (considering the different size datasets).

In fact, the two datasets were collected almost a year apart, in the meantime, the Glacios camera was updated to Falcon4i (hence the difference in dataset size due to the new camera performance), and the data was analyzed by two operators with their own software preferences.
Ultimately, we think that collecting a new dataset of similar size with the B/NP_∆64_ RNP-like particles would yield the same – or highly similar – cryoEM map resolution than the B/NP_∆68_ one, as stated in the discussion line 256-260.
