# Supplementary material for: Nucleocapsid-like cryo-EM structure of influenza B virus
Source: PLoS Pathog. 2026 Aug 10;22(8):e1014449. doi: 10.1371/journal.ppat.1014449 (PMC13497295; doi:10.1371/journal.ppat.1014449)
Supplement: Peer review history

# Peer review: Decision Letter 0

**DOI:** 10.1371/journal.ppat.1014449.r001

**Authors:** Matthias Schnell, Benjamin Hale

14 Jun 2026

PPATHOGENS-D-26-01054

Nucleocapsid-like cryoEM structure of influenza B virus

PLOS Pathogens

Dear Dr. Ballandras-Colas,

Thank you for submitting your manuscript to PLOS Pathogens. After careful consideration, we feel that it has merit but does not fully meet PLOS Pathogens's publication criteria as it currently stands. Therefore, we invite you to submit a revised version of the manuscript that addresses the points raised during the review process.

Please submit your revised manuscript by Jul 14 2026 11:59PM. If you will need more time than this to complete your revisions, please reply to this message or contact the journal office at plospathogens@plos.org. When you're ready to submit your revision, log on to https://www.editorialmanager.com/ppathogens/ and select the 'Submissions Needing Revision' folder to locate your manuscript file.

Please include the following items when submitting your revised manuscript:

* A letter that responds to each point raised by the editor and reviewer(s). You should upload this letter as a separate file labeled 'Response to Reviewers'. This file does not need to include responses to any formatting updates and technical items listed in the 'Journal Requirements' section below.

* A marked-up copy of your manuscript that highlights changes made to the original version. You should upload this as a separate file labeled 'Revised Manuscript with Track Changes'.

* An unmarked version of your revised paper without tracked changes. You should upload this as a separate file labeled 'Manuscript'.

If you would like to make changes to your financial disclosure, competing interests statement, or data availability statement, please make these updates within the submission form at the time of resubmission. Guidelines for resubmitting your figure files are available below the reviewer comments at the end of this letter.

As the corresponding author, your ORCID iD is verified in the submission system and will appear in the published article. PLOS supports the use of ORCID, and we encourage all coauthors to register for an ORCID iD and use it as well. Please encourage your coauthors to verify their ORCID iD within the submission system before final acceptance, as unverified ORCID iDs will not appear in the published article. Only the individual author can complete the verification step; PLOS staff cannot verify ORCID iDs on behalf of authors.

We look forward to receiving your revised manuscript.

Kind regards,

Benjamin G Hale, PhD

Academic Editor

PLOS Pathogens

Matthias Schnell

Section Editor

PLOS Pathogens

Sumita Bhaduri-McIntosh

Editor-in-Chief

PLOS Pathogens

orcid.org/0000-0003-2946-9497

Michael Malim

Editor-in-Chief

PLOS Pathogens

orcid.org/0000-0002-7699-2064

**Additional Editor Comments (if provided):**

Both reviewers are very positive about the manuscript, and both comment favourably about its contribution to the influenza virus literature. The issues raised by the reviewers can all likely be addressed with changes to the manuscript text. Please address all of the issues raised in any revised manuscript.

**Journal Requirements:**

If the reviewer comments include a recommendation to cite specific previously published works, please review and evaluate these publications to determine whether they are relevant and should be cited. There is no requirement to cite these works unless the editor has indicated otherwise.

1) We ask that a manuscript source file is provided at Revision. Please upload your manuscript file as a .doc, .docx, .rtf or .tex. If you are providing a .tex file, please upload it under the item type u2018LaTeX Source Fileu2019 and leave your .pdf version as the item type u2018Manuscriptu2019.

2) We do not publish any copyright or trademark symbols that usually accompany proprietary names, eg ©,  ®, or TM  (e.g. next to drug or reagent names). Therefore please remove all instances of trademark/copyright symbols throughout the text, including:

- ® on pages: 13, 14, and 16

- TM on page: 14.

3) We have noticed that you have uploaded Supporting Information files, but you have not included a list of legends. Please add a full list of legends for your Supporting Information files after the references list.

**Reviewers' Comments:**

Reviewer's Responses to Questions

**Part I - Summary**

Please use this section to discuss strengths/weaknesses of study, novelty/significance, general execution and scholarship.

Reviewer #1: The manuscript by Thirion et al. describes the biochemical and structural characterization of influenza B virus NP-RNA assemblies reconstituted in vitro using truncated NP constructs lacking the long N-terminal disordered tail. The authors show that these truncated proteins retain RNA-binding activity and assemble into right-handed antiparallel helical RNP-like particles whose cryo-EM structure resembles previously reported influenza A and D virus nucleocapsids.

Overall, the manuscript is well written and the conclusions are generally supported by the data. The work provides a useful extension of recent structural studies of influenza virus RNP organization to influenza B virus. While the conceptual novelty is somewhat limited, the study contributes valuable comparative structural information across influenza virus types.

Reviewer #2: Thirion et al describe the results of their structural analysis of an influenza B virus RNP-like particle formed by the in vitro reconstitution of NP-RNA. Helical RNP-like particles were only formed when the N-terminal 64 amino-acid residues were removed from the construct. Full-length WT NP was unable to form helical RNP-like assemblies, forming smaller particles instead. RNA binding by the WT and truncated NP was evaluated by fluorescence anisotropy measurements, yielding Kd values that were broadly consistent across all constructs, suggesting that removal of the N-terminal sequence did not impact RNA binding. Negative stain EM of the in vitro reconstituted RNP-like particles showed that they were structurally similar to purified IBV RNPs.

Cryo-EM and 3D image reconstruction of the RNP-like particles with local/helical symmetry imposition let to intermediate resolution maps at 4.1 Å and 5.3 Å resolution for the two truncated NP-RNA complexes. Guided by the previous X-ray structures for IBV NP and prior IAV and IDV cryo-EM structures, a model was assembled into the higher resolution map, aided by the AI map-modification tool EM-ready.

The resulting model provides insights into the oligomerisation of NP – showing for example how the oligomerisation domain forms a loop that inserts into the neighbouring NP protomer, and how the RNA is routed.

Overall the work appears to be expertly performed and in spite of the challenging target some nice maps have been achieved in which the secondary structure elements are well-resolved lending confidence to the interpretation. Considerable detail is presented concerning side-chain interactions both at protein-protein interfaces and in interactions with the RNA. Given the modest resolution, these side-chain placements are presumably heavily guided by prior high-resolution X-ray data and by the most plausible chemistry. Some caution is warranted here, especially as the map has been subjected to a machine-learning map modification routine (although I note that the phenix refinement was performed using the unmodified map).

Although the discussion focusses mainly on what can be seen in the data, throughout the text it is repeatedly asserted that the findings indicate that the N-terminal disordered region does not ‘participate in the helical architecture’ – and some statements around this are a little difficult to understand. Briefly the absence of the N-terminal region allowed formation of extended helical RNP-like assemblies, while its presence prevented that. The assertion that this region is not important for defining the architecture of the helical RNP seems unfounded then. The main finding that the B/NP-RNA complex is structurally very similar to the previously studied A and D RNPs is on far more solid ground. The disordered region however may profoundly impact the higher-order assembly. This could only really be resolved by 3D reconstruction of the authentic RNP, which might shed light on how the disordered region is accommodated to enable formation of the helical RNP, something that may only be possible through replication-coupled RNP assembly.

**********

**Part II – Major Issues: Key Experiments Required for Acceptance**

Please use this section to detail the key new experiments or modifications of existing experiments that should be absolutely required to validate study conclusions.

Generally, there should be no more than 3 such required experiments or major modifications for a "Major Revision" recommendation. If more than 3 experiments are necessary to validate the study conclusions, then you are encouraged to recommend "Reject".

Reviewer #1: 1. The main structural conclusion that influenza B RNP-like particles adopt a right-handed antiparallel helical organization similar to influenza A and D vRNPs is consistent with recent studies on influenza A and D virus nucleocapsids. However, the discussion should better acknowledge that the major principles of NP-RNA organization and helical assembly were already established in Chenavier et al., Sci Adv 2023; Chenavier et al., NAR 2025; Peng et al., Science 2025.

2. The conclusion that the B/NP N-terminal tail “does not participate to the helical architecture” should be softened. The data convincingly show that truncated proteins can assemble into helical particles and that the tail is not required for RNA binding in vitro or for the formation of RNP-like architecture under the tested conditions. However, it cannot be ruled out that the tail could plays non architectural role in native vRNPs essential to its proper helical assembly. This is especially important given the proposed flexibility and potential regulatory functions discussed later in the manuscript. I suggest rephrasing this conclusion more cautiously throughout the manuscript.

3. The statement that the reconstituted particles “appear identical to vRNPs extracted from viruses” is overstated. The comparison with native B/vRNPs relies mainly on negative-stain EM morphology. While the resemblance is convincing at that level, the difficulties of obtaining high resolution structure from native vRNPS suggest some intrinsic structural differences. I recommend moderating the wording accordingly.

4. The authors appropriately acknowledge that the RNA density is not sufficiently resolved for de novo modelling. However, this limitation should also be extended to the interpretation of the protein model more generally, as the density displays relatively few well-resolved side chains. At this resolution, the map primarily supports rigid-body or flexible fitting rather than detailed atomic interpretation. Consequently, residue-level interactions should be interpreted cautiously. Nevertheless, I think the cryo-EM density remains sufficiently interpretable to support the main conclusions of the manuscript, especially since several of the interactions discussed (for example those described in Fig. 3C) are consistent with previously reported influenza NP crystal structures.

5. The manuscript still lists placeholder accession numbers (“XXX, YYY, ZZZ”) in the Data Availability section and table 1. These must be completed before publication.

Reviewer #2: (No Response)

**********

**Part III – Minor Issues: Editorial and Data Presentation Modifications**

Please use this section for editorial suggestions as well as relatively minor modifications of existing data that would enhance clarity.

Reviewer #1: 1. The authors may consider clarifying why the full-length B/NP fails to assemble into RNP-like filaments in vitro despite native vRNPs obviously containing full-length NP. A short discussion of possible steric or kinetic effects of the disordered tail during in vitro assembly would strengthen the interpretation.

2. Please ensure all supplementary figures and tables are appropriately referenced and described in the main text.

Reviewer #2: Throughout the text the use of B/RNP-like is an adjective without a noun – I suggest B/RNP-like particles, or B/RNP-like complexes.

Line 41. “extended N-terminal tail does not imper RNA binding ability and oligomeric state…” presumably the intention is ‘impair’ however while it doesn’t appear to have a material effect on RNA binding, it certainly does impact the oligomeric state.

Figure 1C – the negative stain EM of the monomeric NP is difficult to interpret – presumably the white arrows indicate a random selection of the particles, but the image is of a field of particles, why use arrows? – are they pointing to a particular subset? The images are not very convincing concerning the homogeneity/monodispersity of the preparation as the particles are so small. Perhaps other methods may be more helpful, such as dynamic light-scattering or mass photometry (if they are available). The gel-filtration shows a reasonably tight-elution profile but would benefit from e.g. silver stained gel to show protein purity.

Line 146 “Inspection of the micrographs confirmed B/NPWT inability to form helical filaments” why confirmed? Was this information already known?

Line 208 “… we observe a density inconsistent attributable to the RNA in both helical strands”. I am not sure what the intention is here – is the density inconsistent with being RNA? Or is the density weak or discontinuous?

Cryo-EM image processing methods. I note that the authors have used a more or less identical approach to determining the structure of the two N-terminally truncated NP-RNA complexes but implemented in Relion for one map and in CryoSPARC for the other. Is there a reason for this? Was the analysis performed by two operators with their own preferences, or did one software yield a better outcome (considering the different size datasets).

**********

PLOS authors have the option to publish the peer review history of their article (what does this mean?). If published, this will include your full peer review and any attached files.

If you choose “no”, your identity will remain anonymous but your review may still be made public.

**Do you want your identity to be public for this peer review?**  For information about this choice, including consent withdrawal, please see our Privacy Policy.

Reviewer #1: **Yes:** Loïc Carrique

Reviewer #2: No

[NOTE: If reviewer comments were submitted as an attachment file, they will be attached to this email and accessible via the submission site. Please log into your account, locate the manuscript record, and check for the action link "View Attachments". If this link does not appear, there are no attachment files.]

**Figure resubmission:**

**Reproducibility:**

To enhance the reproducibility of your results, we recommend that authors of applicable studies deposit laboratory protocols in protocols.io, where a protocol can be assigned its own identifier (DOI) such that it can be cited independently in the future. Additionally, PLOS ONE offers an option to publish peer-reviewed clinical study protocols. Read more information on sharing protocols at https://plos.org/protocols?utm_medium=editorial-email&utm_source=authorletters&utm_campaign=protocols

# Author comment: Author response to Decision Letter 1

**DOI:** 10.1371/journal.ppat.1014449.r002

1 Jul 2026

Attachment Submitted filename: Response to Reviewers_260630.docx

# Editor report: Decision Letter 1

**DOI:** 10.1371/journal.ppat.1014449.r003

**Authors:** Matthias Schnell, Benjamin Hale

6 Jul 2026

Dear Dr Ballandras-Colas,

We are pleased to inform you that your manuscript 'Nucleocapsid-like cryoEM structure of influenza B virus' has been provisionally accepted for publication in PLOS Pathogens.

Before your manuscript can be formally accepted you will need to complete some formatting changes, which you will receive in a follow up email. A member of our team will be in touch with a set of requests.

Please note that your manuscript will not be scheduled for publication until you have made the required changes, so a swift response is appreciated.

IMPORTANT: The editorial review process is now complete. PLOS will only permit corrections to spelling, formatting or significant scientific errors from this point onwards. Requests for major changes, or any which affect the scientific understanding of your work, will cause delays to the publication date of your manuscript.

Should you, your institution's press office or the journal office choose to press release your paper, you will automatically be opted out of early publication. We ask that you notify us now if you or your institution is planning to press release the article. All press must be co-ordinated with PLOS.

Thank you again for supporting Open Access publishing; we are looking forward to publishing your work in PLOS Pathogens.

Best regards,

Benjamin G Hale, PhD

Academic Editor

PLOS Pathogens

Matthias Schnell

Section Editor

PLOS Pathogens

Sumita Bhaduri-McIntosh

Editor-in-Chief

PLOS Pathogens

orcid.org/0000-0003-2946-9497

Michael Malim

Editor-in-Chief

PLOS Pathogens

orcid.org/0000-0002-7699-2064

***********************************************************

Thank-you for addressing all of the comments from the original reviews. I have reviewed the changes and am happy with all of the edits and clarifications made. Thank-you for submitting your work to PLOS Pathogens.

Reviewer Comments (if any, and for reference):

# Editor report: Acceptance letter

**DOI:** 10.1371/journal.ppat.1014449.r004

**Authors:** Matthias Schnell, Benjamin Hale

Dear Dr Ballandras-Colas,

We are delighted to inform you that your manuscript, "Nucleocapsid-like cryoEM structure of influenza B virus," has been formally accepted for publication in PLOS Pathogens.

We have now passed your article onto the PLOS Production Department who will complete the rest of the pre-publication process. All authors will receive a confirmation email upon publication.

The corresponding author will soon be receiving a typeset proof for review, to ensure errors have not been introduced during production. Please review the PDF proof of your manuscript carefully, as this is the last chance to correct any scientific or type-setting errors. Please note that major changes, or those which affect the scientific understanding of the work, will likely cause delays to the publication date of your manuscript. Note: Proofs for Front Matter articles (Pearls, Reviews, Opinions, etc...) are generated on a different schedule and may not be made available as quickly.

Soon after your final files are uploaded, the early version of your manuscript, if you opted to have an early version of your article, will be published online. The date of the early version will be your article's publication date. The final article will be published to the same URL, and all versions of the paper will be accessible to readers.

For Research Articles, you will receive an invoice from PLOS for your publication fee after your manuscript has reached the completed accept phase. If you receive an email requesting payment before acceptance or for any other service, this may be a phishing scheme. Learn how to identify phishing emails and protect your accounts at https://explore.plos.org/phishing.

Thank you again for supporting open-access publishing; we are looking forward to publishing your work in PLOS Pathogens.

Best regards,

Sumita Bhaduri-McIntosh

Editor-in-Chief

PLOS Pathogens

orcid.org/0000-0003-2946-9497

Michael Malim

Editor-in-Chief

PLOS Pathogens

orcid.org/0000-0002-7699-2064
